# A Critical Review of the Molecular and Clinical Effects of Cilostazol After Percutaneous Coronary Intervention

**DOI:** 10.3390/jcdd13010031

**Published:** 2026-01-05

**Authors:** Roberto Ferrari, Pasquale Perrone Filardi

**Affiliations:** 1 European Heart for Children, Corso Ercole I° D’Este 32, 44121 Ferrara, Italy; 2 Department of Advanced Biomedical Sciences, Federico II University of Naples, Via Pansini, 5, 80131 Naples, Italy

**Keywords:** cilostazol, coronary restenosis, phosphodiesterase inhibitor, antiplatelet therapy, drug-eluting stents

## Abstract

**Background**: Restenosis after coronary stent implantation remains a major clinical challenge, especially in patients with diabetes, long lesions, or multiple stents. Standard therapy with aspirin and P2Y12 inhibitors does not reliably prevent this complication. **Objectives**: We reviewed experimental and clinical evidence on cilostazol, a selective phosphodiesterase-3 inhibitor, as a strategy to reduce restenosis after percutaneous coronary intervention (PCI). **Methods**: Preclinical and clinical studies were critically appraised, focusing on the effects of cilostazol on vascular smooth muscle and endothelial cells, platelet aggregation, lipid metabolism, and restenosis rates. **Results**: Experimental models show that cilostazol inhibits smooth muscle proliferation and intimal hyperplasia after arterial injury. Clinical trials demonstrate reduced restenosis after balloon angioplasty and stent implantation compared with aspirin, ticlopidine, or clopidogrel. Although approved by the FDA for intermittent claudication, cilostazol remains underused in the prevention of coronary restenosis. **Conclusions**: Current evidence supports cilostazol as an effective adjunctive therapy to reduce restenosis following PCI. Wider adoption and further large-scale trials are warranted to better define its role in contemporary interventional practice.

## 1. Introduction

Percutaneous Coronary Interventions (PCI) are a widely used procedure for the treatment of coronary artery diseases (CAD). Since the first application in 1977, PCIs have significantly evolved. Bare metal stents (BMS) have overcome the limitation of balloon angioplasty and drug eluting stent (DES), the limitation of BMS. Indeed, this is a story of great success, but not free from recurrences, such as coronary restenosis or, more often, coronary intrastent restenosis, often leading to target lesion revascularisation [1]. This is because insertion of a stent in the coronary arteries causes several injuries, such as reactive smooth muscle cell proliferation and hypertrophy with the development of neointimal hyperplasia and induction of neoatherosclerosis, both being the most important substrates for restenosis. Combination of the metal stent with a polymer-releasing antiproliferative drug (Drug Eluting Stent, DES) has significantly reduced, but not avoided the risk of restenosis, which still occurs at a rate of 1–5% per year even with the most contemporary DES [2]. Thus, prevention of restenosis plays a pivotal role in the long-lasting benefits of PCI.

Different approaches to prevent or avoid restenosis have been considered, from intravascular brachytherapy to intravascular lithotripsy [1]. Nevertheless, dual antiplatelet therapy (DAPT) with aspirin and P2YI2 inhibitor (clopidogrel, prasugrel, or ticagrelor) for 12 months after PCI with DES or 1 month after PCI with BMS remains the gold standard pharmacological treatment. However, DAPT may not be sufficient to prevent coronary thrombosis and restenosis in some patients, especially those with high-risk features, such as diabetes mellitus, long lesions, small vessels, bifurcation, or chronic total occlusions [1]. Moreover, DAPT may increase the risk of bleeding complications, which can also adversely affect the prognosis.

Cilostazol (6-[4-(1-cyclohexyl-1H-tetrazol-5-yl)butoxy]-3,4-dihydro-2[1H]-quinolinone) is a quinolinone derivative and a phosphodiesterase type III inhibitor (PDS3), developed in Japan and approved by the US Food and Drug Administration (FDA) for the treatment of intermittent claudication [3,4]. It exerts both antiplatelet and antiproliferative effects, plus other pleiotropic effects on the cardiovascular system. Cilostazol is not included in the ESC and AHA guidelines. Therefore, it is not used as post-PCI standard therapy in the Western world despite a large literature on its beneficial effects post-PCI [5,6,7,8,9,10,11,12,13,14,15,16,17,18,19,20,21,22,23,24,25]. However, it is an old drug that may be another option to reduce coronary restenosis and further improve clinical outcomes in patients undergoing PCI [2].

We conducted a systematic review of the literature limited to the effects of cilostazol on post-PCI restenosis, including its mechanism of action, pharmacology, and clinical evidence. To this end, we have analysed all the titles and abstracts in Scopus, PubMed, and Google Scholar containing the keywords “cilostazol” and “restenosis”. Our review does not take into consideration data and results pertaining to the effects of cilostazol in peripheral artery diseases, stroke, or carotid artery restenosis, as these topics have been recently covered in an excellent comprehensive review by Manolis et al., to which interested readers are addressed [5].

The aim of our manuscript is to provide the readers with a concise way of understanding the effects of cilostazol in post-PCI restenosis, despite the fact that the molecule is not indicated after PCI, most likely because of the lack of interest of the cilostazol producers to enter a highly competitive market. However, this does not diminish the scientific interest in its effectiveness.

## 2. The Contests: POST-PCI Coronary Restenosis

Angiographic restenosis is defined as a binary angiographic luminal narrowing of a coronary artery greater than 50%, either within the stent (in-stent restenosis) or outside the stent, including 5 mm proximal or distal to the stent margin (in segmental restenosis). Angiographic restenosis can also be measured in terms of late loss, which is the difference in mm between the minimal luminal diameter (MLD) of the lesion immediately after PCI and the MLD at follow-up angiography [1].

The pathophysiologic prerequisite of restenosis is the development of further obstructive CAD, generating (not always) ischaemic symptoms, i.e., exertional angina or unstable angina as a result of myocardial underperfusion. However, the first presentation of restenosis is often an acute coronary syndrome and myocardial infarction [1]. Presence of these symptoms after a successful PCI describes a condition known as “clinical restenosis”. Clinical restenosis occurs with a luminal renarrowing greater than 50% in the presence of symptoms of ischaemia or in the absence of symptoms but with an abnormal result of invasive testing, such as IVUS (<16 mm for the left main coronary artery and <4 mm for the others) or FFR < 0.80. Usually, angiographic restenosis is more frequent than clinical restenosis.

Several different mechanisms are responsible for the development and the severity of coronary restenosis. These include patients’ related characteristics, biological and anatomic features, and procedural or stent factors.

Age, female sex, obesity, diabetes, and kidney diseases are all clinical predictors of restenosis. Among these, diabetes is the most important as the metabolic alterations consequent to hyperglycaemia and hyperinsulinemia accelerate the formation of neointimal hyperplasia. In general, the process of restenosis includes (1) neointimal hyperplasia caused by migration and proliferation of smooth muscle cells with production of extracellular matrix, (2) elastic recoil of the vessel after balloon inflation with negative remodelling of the vessel, and, eventually, (3) plaque deposition and aggregation with formation of a thrombus. Biological factors favouring restenosis include either primitive (genetic) or secondary resistance to antiproliferative and antiaggregant intervention. Often, there is an excessive reaction to a metallic or polymeric platform, causing endothelial denudation with pronounced and persistent inflammation with leukocyte infiltration, fibrin deposition, and delayed re-endothelialisation. This series of events usually occurs more often with the first generation of BMS, whilst in the second-generation DES, there is faster re-endothelialisation and less fibrin deposition. There are major differences between BMS and DES. With BMS, the restriction of the lumen of the coronary artery (expression of neointimal accumulation) tends to occur within 6–8 weeks, whilst with DES, this process (when it happens) is slow and might occur over the years after implantation. Regardless of the cause and timing of occurrence, excessive proliferation of smooth muscle cells embedded in a collagen-rich extracellular matrix is one of the most common pathological features of restenosis. Different histopathological lesions have been described. Restenosis of BMS is characterised by homogeneous, dense smooth muscle cell proliferation, with a close association between the extent of medial damage, inflammation, leukocyte infiltration, and restenosis [26]. After DES, hypocellular contractile cells are more common [27]. Post-mortem studies with first-generation DES show a greater persistence of inflammation, delayed endothelialisation, and arterial healing compared to BMS. Restenosis after the second generation of DES was associated with less fibrin deposition, showed more uniform endothelialisation, and less inflammation compared to BMS and first-generation DES [28]. Another typical feature of late restenosis after DES is the formation of intra-stent neoatherosclerosis, characterised by the accumulation of lipid-loaded macrophages with or without calcification and necrotic core. This represents a sort of accelerated atherosclerosis due to insufficient endothelial coverage and occurs late or very late [28].

Procedural aspects are also important determinants of restenosis, and correct stent implantation is of paramount importance to avoid restenosis. The most frequent cause of restenosis is stent malposition, under-expansion, as well as stent gaps, leaving discontinuous coverage of a coronary lesion between two stents. Among the stent-related factors, the most frequent relate to stent typology, stent thickness, the type of antiproliferative drug used, method of release and distribution, and, of course, stent fracture. The size of the vessel, characteristics of the lesion, and the degree of shear stress are all factors that can influence and, at the same time, predict restenosis. Small vessels, calcified lesions, and high thrombus burden all favour restenosis.

Based on randomised control trials, rate of angiographic restenosis after balloon ranges between 32 and 42% per year, and half of these patients require clinically driven target lesion revascularisation within the first year. Stenting the coronary artery prevents the negative remodelling of the vessel and the elastic recoil, but as previously pointed out, it might cause neointimal hyperplasia as a result of smooth muscle cell proliferation and production of extracellular matrix. With BMS, angiography restenosis rates typically occur within the first 6 months, with a rate between 16% and 32%, and the need for clinically driven target lesion revascularisation in about 12% of cases per year. Rates of restenosis with DES are lower, ranging from 1 to 5% per year depending on the type of DES, morphology of the lesion, and characteristics of the patient. Compared with BMS, DES significantly reduced the need for target lesion revascularisation with a lower effect on all-cause mortality. Although the rate of restenosis with the most sophisticated contemporary DES platform might be considered acceptable, it should not be underestimated, as millions of DES are implanted every year worldwide, thus leading to a huge amount of restenosis that needs to be treated with all the related costs [1]. It follows that restenosis is still considered a pathologic entity of public health significance. About 10% of total PCIs in the United States are performed to treat restenosis and are associated with a higher risk of major cardiac events compared to PCI of de novo lesions [29]. Therefore, the global burden of coronary restenosis remains an important clinical problem, even in the face of the most advanced DES platform. Pharmacological prevention of restenosis and of in-stent thrombosis is of paramount importance for the long-lasting benefits of PTCI.

So far, accumulated experience suggests that cilostazol could be a useful, although underutilised, agent to be co-administered with double antiplatelet therapy to prevent coronary restenosis and to avoid platelet aggregation in patients with CAD undergoing PTCI. Here, we review the molecular rationale that has supported the clinical studies with cilostazol.

## 3. Molecular Properties of Cilostazol

The main effect of cilostazol is a selective inhibition of cyclic nucleotide phosphodiesterase 3 (*PDE3*), an enzyme member of a genetically distinct family within the 1–11 phosphodiesterases. PDE3 is responsible for the degradation of both cyclic adenosine phosphate (*cAMP*) and cyclic guanosine monophosphate (*cGMP*), the synthesis of which is regulated by adenylate cyclase and guanylate cyclase, respectively. Cilostazol acts on the balance between synthesis and breakdown of cyclic nucleotides, leading to increased levels of intracellular cAMP with relative phosphorylation of several protein kinases and optimisation of cellular calcium movements [6]. In humans, PDE3 activity is expressed in platelets, endothelial cells, smooth muscle cells, cardiac myocytes, and adipocytes. Therefore, the molecular and clinical effects of cilostazol occur in these different districts of the body, and all contribute in various ways to the action of cilostazol on coronary (and not only) restenosis [30]. Cilostazol also inhibits adenosine uptake in vascular cells and myocytes [31]. The consequent increase in interstitial and circulating adenosine enhances the intracellular cAMP activity in platelets and smooth muscle cells, thus potentiating the anti-aggregation and vasodilation induced by cilostazol.

The molecular targets to prevent restenosis include avoiding smooth muscle proliferation, platelet aggregation, and thrombus formation. In addition, to attenuate all these molecular steps leading to restenosis, cilostazol exerts several other actions on the endothelium and smooth muscle cells of the vessels, including the coronaries, and on lipid metabolism, which may also be beneficial. This sort of pleiotropic effect differentiates cilostazol from other antiaggregant drugs and is likely to play an adjuvant role for the prevention of further progression of the coronary artery restenosis.

Effect on smooth cell proliferation: Neointimal formation, due to proliferation of vascular smooth muscle cells (*VSMCs*), is the leading cause of restenosis in response to arterial injury [32]. This is particularly so in diabetic patients who have higher restenosis rates than non-diabetic patients. Several lines of evidence suggest that cilostazol is effective in attenuating VSMCs and neointimal proliferation.

In vitro, cilostazol specifically counteracts the proliferation of both rats’ and humans’ smooth muscle cells, induced by a variety of growth factors such as insulin-like growth factor 1 or platelet-derived growth factor (*PDGF*) [33,34]. Most likely, this action depends upon cilostazol-induced cAMP in the smooth muscle of the coronary arteries, which inhibits cell growth by inhibiting mitogen-activated protein kinases and reducing the release of PDGF. Such a reduced availability of PDGF also plays an important role in the inhibition of smooth muscle cell proliferation. Of note, the anti-proliferation effect of cilostazol is specific for the smooth cell, as on isolated endothelial cells, cilostazol induces a cell-specific (endothelial) growth factor [35]. Such dual action, inhibition of growth factors on the smooth muscle cells and activation of growth factors on endothelial cells, is clinically relevant in the context of restenosis. On one hand, cilostazol favours growth of the endothelium, thus increasing endothelialisation of the stent and avoiding further platelet aggregation; on the other hand, it reduces the proliferation of muscular components of the artery, thus attenuating restenosis.

These effects on the endothelium do not occur only in experimental settings, but also in humans. Cilostazol has been reported to increase bone marrow production and mobilisation of endothelial progenitor cells and other angiogenic markers, thus contributing to endothelial repair [7,36]. This effect does not occur with aspirin or clopidogrel, thus attesting to a cilostazol effect not only against restenosis but also against the occurrence of thrombosis. Interestingly, cilostazol improved coronary endothelial dysfunction in patients with vasospastic angina and, consequently, increased coronary blood flow [8].

In vivo experiments with carotid artery ballooning in rats [29] and stenting in dogs [37] demonstrated that cilostazol reduced neointimal hyperplasia and smooth muscle proliferation induced by both procedures. The same effects are also seen in men, as cilostazol inhibits human VSMCs in response to high glucose exposure [32]. Accordingly, cilostazol attenuated the increase in intima-media thickness, a recognised index of atherosclerosis in diabetic patients [38]. These effects are found to be mediated by the cilostazol increase in cAMP leading to a cascade of events, such as (1) upregulation of anti-oncogenes p53 and p21 which, in turn, cause apoptosis of vascular smooth cells; (2) upregulation of hepatocyte growth factor, which also inhibits abnormal smooth muscle cell growth and improves endothelial function; (3) upregulation of HGF, which stimulates regeneration of endothelial cells which, in turn, attenuates neointimal formation; and (4) downregulation of the transcription factor E2F, which leads to the activation of genes needed for cell-cycle completion [32,39,40].

In humans, Ahn et al. showed the antiproliferative effects of cilostazol on carotid media thickness in 141 diabetic patients. Cilostazol administration (110/200 mg/day) for 12 months was well tolerated and significantly reduced the size of left and right common intima (measured by ultrasound), which, on the contrary, increased in placebo-treated patients [41]. These data were confirmed in 41 patients subjected to coronary atherectomy. Either cilostazol or aspirin was administered before atherectomy and continued for 6 months. The minimal lumen diameter at follow-up, determined by the quantitative core, was larger in the cilostazol group than in the aspirin group, confirming the relevant role of cilostazol in coronary restenosis [42]. Inoue et al. [43] tested the hypothesis that cilostazol inhibits restenosis by the inhibition of leukocyte integrin MAG-1, which is upregulated on the surface of neutrophils in association with restenosis. They determined, by flow cytometry of coronary sinus blood, the expression of platelet membrane glycoproteins and neutrophil adhesion molecules in 70 patients undergoing PTCI, randomised to either clopidogrel or cilostazol. Cilostazol prevented restenosis at a better rate than clopidogrel and increased platelet P-selectin, while suppressing neutrophil MAG-1.

When taken together, these data suggest that cilostazol exerts a clear inhibition of vascular smooth cell growth whilst stimulating the growth of endothelium. These effects are expected to prevent neointimal formation and restenosis, as well as to favour endothelialisation of the stent’s platform.

Effects on platelet aggregation: In early animal studies on mice, rabbits, and guinea pigs, cilostazol was shown, by elevating intracellular cAMP and by inhibiting adenosine uptake, to inhibit platelet aggregation elicited by a range of chemicals and physical stimuli [44]. The mode of action of cilostazol is different from that of aspirin. Cilostazol inhibits not only secondary but also primary platelet aggregation due to the effect on adenosine [45]. As a result, cilostazol prevents platelet aggregation induced by both collagen and ADP, while aspirin prevents only aggregation induced by collagen.

In in vitro studies, cilostazol induced a dose-dependent reduction in platelet aggregation, independent of the absence or presence of the endothelium. However, the presence of the endothelium is relevant as cilostazol potentiates the release of the endothelium-derived prostacyclin, which, in turn, inhibits platelet aggregation [46]. Cilostazol is more selective for PDE3 than milrinone or pentoxifylline, inhibits adenosine uptake, and, at the same time, decreases the thromboxane A_2_ production, resulting in a robust and reversible antiplatelet effect as platelet function recovers within 12–16 h after withdrawal [47,48].

In in vivo experiments in mice, cilostazol inhibited ADP and collagen-induced pulmonary thrombosis, resulting in reduced mortality [48]. The same anti-thrombotic effect was confirmed in a porcine carotid artery model and in dogs after thrombolysis achieved by recombinant tissue-type plasminogen activator [49,50]. Cilostazol decreases the risk of haemorrhage vs. other antiplatelet agents [6]. Of note, the antiaggregant effect of cilostazol is confirmed also in diabetic patients in whom it significantly reduced platelet-derived microparticles as well as soluble adhesion molecules [51].

Preliminary explorative test in humans indicates that cilostazol inhibits ADP, norepinephrine, collagen, and arachidonic acid-induced platelet aggregation similarly to aspirin and ticlopidine, respectively, but without any effect on bleeding time, which, on the contrary, is significantly increased by aspirin and ticlopidine [52]. Such effects were confirmed by a study on 21 patients with peripheral arterial disease, showing that the addition of cilostazol to aspirin (300 mg daily) and clopidogrel (75 mg daily) had no significant effect on bleeding time, which was increased by the two antiplatelets [53]. This is relevant as one of the classical “Yin and Yan” dilemmas after angioplasty, which is the equilibrium between prevention of ischaemia by anti-aggregation in the face of an increase in bleeding.

These data support a robust inhibition of platelet activation and aggregation by cilostazol, which, in clinical practice, should translate into inhibition of thrombosis at the site of restenosis.

Effects on the endothelium: Besides stimulating the growth of the endothelium and ameliorating its dysfunction, cilostazol exerts other direct effects on the endothelium. These have been determined by Nakamura et al. in the rat’s thoracic aorta [54]. Cilostazol induced a dose-dependent relaxation of the phenylephrine pre-contracted thoracic aorta. In the same preparation, physical denudation of the endothelium reduced the effects of cilostazol in comparison with the intact (with the endothelium) aortic preparation. This suggests that the relaxant activity of cilostazol is partly, at least, mediated by the endothelium and, more specifically, by a release of nitric oxide (NO) from it. Such a sequence of events is confirmed as the cilostazol-induced relaxation is inhibited by N-Nitro-L-arginine, which, in turn, abrogates the production of NO from nitric oxide synthase. These effects of cilostazol have also been shown in the porcine thoracic aorta and in the aortic ring of rats [32].

In humans, the vasodilation induced by cilostazol has translated into an increased blood flow to the limbs, which supports its indication in the treatment of peripheral arterial disease. In this regard, cilostazol has been studied in 10 trials. It has been shown to improve maximum walking distance and to be superior to pentoxifylline in patients with Raynaud’s phenomenon to the point that it was recently proposed to repurpose cilostazol in this indication in view of its vasodilating and antiplatelet effects [55]. Similar effects are reported for coronary and cerebral arteries, where cilostazol has been shown to effectively reduce vasospasm and improve flow [13,56]. These actions are expected to result in the negative remodelling of the vessel induced by stents’ application and to contribute to preventing restenosis.

Effects on plasma lipids: PDE3, which is inhibited by cilostazol, is expressed in adipocytes. Therefore, it is not surprising that the effects of cilostazol have also been investigated on lipid metabolism. Studies in rats show a cilostazol-induced reduction in plasma triglycerides and an increase in high-density lipoprotein cholesterol (HDL cholesterol), likely due to an enhanced activity of plasma lipoprotein lipase [57]. The same findings have been confirmed in randomised (vs. placebo) clinical studies looking at plasma lipoprotein profile [58]. Active treatment increased HDL cholesterol by 10% and apolipoprotein A1 by 5.7% (*p* < 0.01), whilst reducing triglycerides. Patients with hypertriglyceridemia were those experiencing the biggest reduction in both HDL-C and triglycerides. In the same cohort, there was a trend toward a reduction in apolipoprotein B. Similar results were also obtained in diabetic patients [59].

Although these favourable effects on the pro-atherogenic lipid profile are unlikely to play a direct role in the reduction in restenosis, they definitely cause a more anti-atherogenic profile of the plasma of patients subjected to PTCI and, hopefully, will play a role in the prevention of other coronary plaque formations and maintenance of coronary artery patency.

Other cardiovascular effects not related to restenosis: Cilostazol exerts a positive ionotropic and inotropic effect on the myocardium. Studies with Holter electrocardiogram monitoring suggest some caution, as cilostazol can elevate heart rate [60]. For this reason, cilostazol has been tested in patients with sick sinus syndrome to avoid pacemaker implantation [61]. In this context, cilostazol turned out to be more effective than theophylline [62]. Animal experiments indicate that cilostazol improves left ventricular function after acute myocardial infarction but does not reduce infarct size and may increase the incidence of arrhythmias [63].

Although different from milrinone—another inhibitor of PDE3 that exerts anti-arrhythmic effects in heart failure—cilostazol has been shown to possess antiarrhythmic action [31]. In view of this finding, cilostazol has also been investigated in patients affected by the Brugada syndrome. In these patients, cilostazol reduced the incidence of ventricular fibrillation, probably related to a reduction in the transient outward potassium and/or calcium currents secondary to the increase in cAMP in the myocardium, with no effect on sodium current.

## 4. Pharmacokinetics and Side Effects

The peak absorption of cilostazol occurs two to three hours after its intake. Cilostazol extensively binds to proteins (95%), mainly albumin, and its pharmacokinetic is independent of age and sex.

Cilostazol is metabolised in the liver by cytochrome P450 (CYP) isoenzymes, mainly by CYP3A4 and to a lesser extent by CYP2C19. Thus, cilostazol has two main metabolites, which account for about 50% of PDE3 inhibition [5,14]. Its elimination depends on renal excretion with a half-life of 11–13 h, but it is extended in patients with severe kidney insufficiency.

The recommended dose is 100 mg twice daily, but a reduction to 50 mg two times a day is recommended in case of co-administration with drugs that inhibit CYP3A4 or CYP2C19, such as omeprazole or erythromycin [15]. Aspirin co-administration does not change the platelet anti-aggregation activity or the plasma level of cilostazol. In general, there are great inter-individual fluctuations in the pharmacokinetic values of cilostazol.

The main side effects are headache, diarrhoea, and gastrointestinal complications. A large post-marketing surveillance in the US on patients with intermittent claudication showed only a few cases of serious adverse events (myocardial infarction, stroke, or death), which, when pooling together the data of the several randomised trials, were no more evident than those of placebo [64]. Interestingly, a recent meta-analysis of 18 randomised clinical trials suggests a decreased risk of intracranial bleeding and haemorrhage in comparison with aspirin and clopidogrel.

In summary, it seems that cilostazol has a very safe profile, with only a few minor side effects. Caution requiring a dose reduction to 50 mg twice daily should be considered in case of severe hepatic and kidney insufficiency and with concomitant administration with agents that inhibit CYP3A4 or CYP2C19.

## 5. Clinical Literature Review

Cilostazol has been tested in patients with CAD subjected to PCI with balloon angioplasty and/or BMS or DES implantation, often as part of DAPT or triple therapy (TAPT). The doses that have been mostly used are 200 mg as a loading dose, followed by 50 to 100 mg as maintenance.

The effects of cilostazol have been compared with those of clopidogrel. Lee et al. investigated the safety and effectiveness of cilostazol vs. clopidogrel in 689 patients who received bare metal stents, randomly assigned to the two groups, plus aspirin [65]. There were similar incidences of all the endpoints, including intrastent thrombosis. The ACCEL-DM study (adjunctive cilostazol vs. double-dose clopidogrel in diabetes mellitus) randomised diabetic patients in need of a PTCI with BMS to a triple group, receiving cilostazol, aspirin, and clopidogrel, to a double group who took a double dose of clopidogrel (150 mg a day) plus aspirin for 30 days. The results show that adding cilostazol causes greater platelet inhibition than doubling the dose of clopidogrel. Of note, this happened in diabetic patients not affected by genetic polymorphism for CYP. On the other hand, the ACCEL-loading ACS, which followed the same protocol of ACCEL-DM but enrolled patients with an acute coronary syndrome, failed to show the same benefit. The frequency of clinically serious bleeding and adverse events was similar between groups in both studies.

These comparative data with clopidogrel encouraged a series of studies, which will be described later, comparing DAPT with clopidogrel and aspirin to TAPT, a combination of clopidogrel, aspirin, and cilostazol.

Cilostazol has also been considered a replacement for clopidogrel in patients hypo-responsive or intolerant to clopidogrel [66,67]. Cilostazol has been proven to counteract clopidogrel resistance in patients undergoing PCI with DES [16]. The CREATIVE trial showed that in patients diagnosed as resistant to clopidogrel by thromboelastography and receiving a DES, addition of cilostazol to intensify platelet anti-aggregation improved all clinical outcomes without increasing the risk of bleeding [68]. Therefore, cilostazol has been considered as a replacement for either clopidogrel or ticlopidine in case of adverse reaction to both drugs [69,70], or in case of clopidogrel-induced neutropenia [17,71]. Cilostazol may also be a useful substitute for clopidogrel in stent-treated patients with a CYP2C19 loss-of-function allele [72]. A study has evaluated the value of cilostazol in aspirin-resistant patients by measuring the antiplatelet activities of sequential intake of cilostazol. Sequential administration of cilostazol up to 200 mg to 300 mg of aspirin in these resistant patients led to a decrease in the number of non-responders from 45.5% to 12.2% [73].

Studies with bare metal stenting: The first studies, either as monotherapy or, later, in combination with aspirin, showed that cilostazol is a safe and efficacious antiplatelet and anti-restenosis agent in this setting [5,74]. Among the earlier studies, one of the most relevant is the CREST trial (cilostazol for restenosis trial). CREST is a multicentric, randomised, double-blind, placebo-controlled trial comparing triple therapy (aspirin, clopidogrel, and cilostazol) vs. double therapy (aspirin, clopidogrel) in 705 patients who received BMS [75]. Cilostazol was given at 100 mg twice daily for six months. Six months after PCI, the luminal diameter of the coronary artery was significantly greater in the group receiving cilostazol, as was the late lumen loss, suggesting less neointimal hyperplasia after coronary stenting. A post hoc pre-specified analysis suggests that the high-risk patients identified in terms of diabetes, small vessels, long lesions, and Left Anterior Descending (LAD) coronary artery lesions achieved the best benefit from the addition of cilostazol to the usual DAPT. There was no difference between groups in terms of major bleeding, subacute stent thrombosis, target vessel revascularisation, stroke, or myocardial infarction. A much larger study by Lee et al. evaluated TAPT with cilostazol versus DAPT with aspirin and clopidogrel in 3012 patients receiving BMS. There were no major changes after six months between groups, with the exception of the incidence of stent thrombosis, which was lower in the cilostazol group. However, the study was open-label [76].

Another study compared cilostazol vs. aspirin in a small group of patients receiving BMS [77]. At 6 months, the minimal lumen diameter was significantly larger (*p* < 0.001) in the cilostazol vs. aspirin group [18].

In summary, these early studies suggest that cilostazol is a safe and effective antiplatelet agent and point to a possible reduction in the rate of restenosis induced by BMS implantation.

Studies with drug-eluting stents: The efficacy of cilostazol to reduce restenosis in patients treated with DES was evaluated in a series of randomised control trials: the DECLARE studies (drug-eluting stenting followed by cilostazol treatment reduces late restenosis). DECLARE examined two groups of patients at high risk for coronary restenosis: those with long coronary stenoses and those with diabetes. In DECLARE-LONG, patients were randomised to either TAPT with aspirin, clopidogrel, and cilostazol or DAPT without cilostazol [19]. In the 500 patients with long coronary artery lesions (≥25 mm) implanted with DES, after 6-month treatment, cilostazol significantly reduced (*p* < 0.001) the primary endpoint, which was the in-stent late loss, a surrogate marker of neointimal hyperplasia. The other secondary endpoints, such as death (0% vs. 0.8%, *p* = 0.499), myocardial infarction (3.6% vs. 8.0%, *p* = 0.036), and MACE (2.8 vs. 7.6%, *p* = 0.066), were significantly lower in the TAPT group. Incidence of bleeding was similar between the two groups. A word of caution is needed for interpreting these results in consideration of the open-label nature of the trial and of its relatively short duration, which could underestimate the time for restenosis.

The design for DECLARE-DIABATES was similar to DECLARE-LONG [20]. A total of 400 diabetic patients (mean haemoglobin A_ic_ 7.7%) with angina or an acute coronary syndrome, subjected to DES implantation (64% with multivessel disease), were randomised to TAPT with the addition of cilostazol or DAPT with aspirin and clopidogrel only. The TAPT group experienced significantly less in-stent late loss (0.25 ± 0.53 vs. 0.38 ± 0.54 mm; *p* = 0.0025). Within the secondary endpoints, in the cilostazol group versus the DAT group, there was a decreased risk of in-segment restenosis at 6 months and of target lesion revascularisation at 9 months. As for DECLARE-LONG, bleeding risk was similar between groups, but drug discontinuation was greater in the TAPT group. The same limitations toward DECLARE-LONG are also valid for DECLARE-DIABETES. At least one of the limitations related to the too short follow-up period (6 to 9 months) is effectively addressed by the analysis of both DECLARE studies after 2 years. At 2 years, patients who received triple antiplatelet therapy (TAPT) showed less need for revascularization of the target lesions than those receiving double antiplatelet therapy (DAPT). In the TAPT group, there was also a significant (*p* = 0.007) reduction in MACE and of the combined outcome of death and myocardial infarction (*p* = 0.007). Even at 2 years, there were no differences in minor or major bleeding between the 2 groups. Thus, the main limitations remain the open-label design and the small number of patients involved, but the results of both DECLARE studies are consistent and point to a benefit of cilostazol addition to usual DAPT in high-risk patients to avoid restenosis after DES implantation. Interestingly, there was an important interaction between the type of stent implanted and antiplatelet therapy. The reduction in risk of restenosis was most obvious with the use of sirolimus-eluting stents in comparison to the use of paclitaxel-eluting stents [21]. The same post hoc analysis showed that the benefit of triple therapy was more evident when the length of the stent exceeded 40 mm.

On the contrary, no difference, in terms of MACE, between TAPT with cilostazol and DAPT was found in a registry of more than 660 patients receiving either everolimus- or zotarolimus-eluting stents. This was the COACT study (Catholic Medical Centre Percutaneous Coronary Intervention) [78]. Another registry (DECREASE PCI) evaluated the influence of TAPT with cilostazol versus DAPT on patients receiving second-generation DES. There was a clear benefit after one year of therapy as the primary endpoint, a composite of mortality, myocardial infarction, stroke, and revascularisation, was significantly less for TAPT [77]. On the other hand, a negative outcome was reported in another randomised trial, the CILON-T (cilostazol-based triple antiplatelet therapy on ischaemic complications after drug-eluting stent) [79]. The authors randomised 960 patients receiving DES to TAPT or DAPT. At 6 months follow-up, there was no difference in the primary composite endpoints (CV mortality, myocardial infarction, ischaemic stroke, and revascularisation). Another negative registry was the INTERSTELLAR registry, which retrospectively analysed 1278 patients receiving DES treated with either TAPT or DAPT [80].

Another later study retrospectively compared the safety and efficacy of TAPT vs. DAPT with aspirin and the newer, more potent PZY12 inhibitor (ticagrelor or prasugrel) in 4152 Korean patients undergoing DES for acute myocardial infarction. After propensity-score matching analysis, there was no difference between groups after one or two years. Of interest, minor and major bleedings were significantly reduced in the TAPT group than in the DAPT group.

Meta-analysis: Several meta-analyses have been conducted on the effects of cilostazol when added to DAPT in patients receiving either BMS or DES. With all the well-known limitations of meta-analysis, the main messages are that cilostazol added to DAPT significantly reduced restenosis and the need for revascularisation, whilst rates of subacute stent thrombosis or major bleeding were not different [6,81]. The pooled data specify that the positive effects of cilostazol on revascularisation do not occur in the short term, but only at mid- or long-term follow-up [82]. Meta-analyses are also consistent in showing that TAPT with cilostazol confers a significant decrease in target lesion and target vessel revascularisation but at the expense of a higher incidence of minor side effects, without a significant increase in bleeding [83,84]. Actually, compared to prasugrel or ticagrelor-based double therapy, administration of cilostazol further decreased bleeding and MACE [22]. We believe that these are the main messages arising from the majority of meta-analyses. For fairness, it is important to underline that the results did vary and sometimes were controversial, depending on when the meta-analysis was performed and on which studies were analysed. This is especially true for MACE or composite endpoints of CV mortality and myocardial infarction, which were reported either unchanged in some meta-analyses or lowered in others [22,23,24]. Interestingly, a meta-analysis specifically assessed platelet reactivity in patients subjected to PCI and found that cilostazol, added to DAPT, reduced platelet reactivity and improved outcomes [85]. Finally, one of the most recent meta-analyses confirmed that triple therapy with cilostazol reduced in-stent stenosis and the need for revascularisation [86].

In summary, the so far accumulated clinical evidence that we have here reviewed on the use of cilostazol in patients with acute or chronic ischaemic heart syndromes subjected to PCI with either balloon or stent implantation provides convincing evidence of cilostazol in preventing restenosis events by exerting a robust antiproliferative action on smooth muscle cells of the coronary arteries, counteracting neointimal hyperplasia. DAPT with aspirin and a P2Y12 receptor antagonist is the actual guideline-recommended standard of care therapy in patients undergoing PCI and stenting. Addition of cilostazol to DAPT with aspirin and ticlopidine has been shown to significantly decrease platelet reactivity and further increase their antiaggregant effects, although interestingly, stenosis was not always further prevented. Today, newer antiplatelet agents, such as prasugrel and ticagrelor, are preferred to clopidogrel (also by the guidelines) as they are more potent and improve further outcomes. Cilostazol has not been tested again on these agents, apart from a few studies with a limited number of patients. Thus, there is a need to explore further the effects of cilostazol in combination with these drugs. Its anti-restenosis effects could be useful and contribute to maintaining stent patency. However, clopidogrel is often preferred to the new P2Y12 for economic reasons or for an increased risk of bleeding. In this case, it is important to underline that comparative studies using TAPT with cilostazol vs. DAPT with aspirin and clopidogrel, and the relative meta-analysis have documented the benefits of cilostazol-added treatment. Finally, cilostazol is a valid alternative in case of aspirin or clopidogrel resistance. Cilostazol also exerts an appreciable action against platelet aggregation, similar to, if not superior to, that of aspirin and ticlopidine.

## 6. Conclusions

The clinical evidence accumulated so far suggests that cilostazol is a safe and potentially useful agent not only for intermittent claudication, for which cilostazol has an FDA indication, but also for CAD, particularly for patients undergoing percutaneous revascularisation. Our comprehensive review of the literature indicates that, when utilised in combination with standard DAPT with aspirin and clopidogrel, it exerts a robust action in preventing restenosis. Less evidence is available for the newer antiaggregants such as prasugrel or ticagrelor. The intimate molecular effects of cilostazol are plausible and explain the mechanism responsible for platelet aggregation. Thus, the biological effects of cilostazol fully support clinical results. However, cilostazol possesses other pleiotropic actions on the cardiovascular system and on lipid metabolism, which might strengthen its effects on restenosis. Despite this evidence, cilostazol is underutilised at least in the Western world. This is probably due to the lack of large randomised clinical trials as are available for other antiaggregants and anti-thrombotic agents. Therefore, individualised risk-benefit assessment and careful monitoring are warranted when considering cilostazol for the treatment of patients after PCI.

## Data Availability

The data will be available upon request.
